# Parametric Formulae for Elastic Stress Concentration Factor at the Weld Toe of Distorted Butt-Welded Joints

**DOI:** 10.3390/ma13010169

**Published:** 2020-01-01

**Authors:** Yuxiao Luo, Renle Ma, Seiichiro Tsutsumi

**Affiliations:** 1 Department of Structural Engineering, Tongji University, Shanghai 200092, China; marenle@tongji.edu.cn; 2 Tongji Architectural Design (Group) Co., Ltd., Shanghai 200092, China; 3 Joining and Welding Research Institute, Osaka University, 11-1 Mihogaoka, Ibaraki, Osaka 567-0047, Japan; tsutsumi@jwri.osaka-u.ac.jp

**Keywords:** butt welds, stress concentration factor, angular distortion, clamping stress, parametric formula

## Abstract

The evaluation of the stress concentration factor (SCF) at the notches of welds is of importance, especially for butt-welded joints that are widespread in the industry. Some empirical formulae can be found in the literature to estimate the SCF at the weld toes of butt-welded joints, while few solutions are available for the distorted joints under tensile fatigue test conditions. In the present study, the existing SCF formulae for butt-welded joints loaded in tension are examined and discussed. The influence of the weld width on SCF, which is commonly ignored or misestimated by existing solutions, is investigated comprehensively based on a large set of two-dimensional (2D) finite element analyses. Consequently, a new precise parametric formula for the elastic SCF at the weld toe of geometrically symmetric butt-welded joints under tension is proposed, together with a wide application range. Moreover, the analysis is also extended to consider joints with angular distortion. A two-step finite element analysis is employed to simulate the clamping and loading procedures in the fatigue test. Similarly, the parametric formulae for the assessment of clamping-induced stress and SCF caused by angular distortion are carried out as well based on the results from finite element analyses. The formulae proposed by this paper are finally tested and proved to be valid and precise.

## 1. Introduction

Welded joints are now extensively used in structures such as bridges, towers, ships, and pressure vessels. This ubiquity highlights the importance of the investigations on the mechanical properties of welds. When subjected to cyclic loading, the failure of these welded joints usually starts from the potential critical spots where discontinuities and notches exist. The fatigue behavior of these welded joints is complicated by a mass of factors, such as the weld profile, the microstructure of the heat-affected zone, and the residual stress around the welds. The influences of the weld geometry and details [1,2], residual stress [3], heat treatment [4,5], and loading type [6] on the fatigue behavior of welded joints have been investigated by the fatigue experimental research. Among all these factors, stress concentration caused by geometric discontinuities and notches is one of the decisive factors that affect the fatigue behavior of welded joints.

This stress concentration effect can be expressed by the stress concentration factor (SCF) which is quite valuable in engineering designs and scientific research. Various local stress-life approaches for fatigue performance assessment are implemented based on the evaluation of SCF at the notches of welds. For example, the effective notch stress approach, which was proposed by Radaj [7] based on Neuber’s stress averaging hypothesis [8], requires the elastic SCF at the fictitious weld toe (toe radius ρ=1.0 mm) to determine the local notch stress, so that fatigue behavior can be assessed with a uniform notch S–N curve [9].

Usually, the profiles of weld beads are varied, and the SCF is influenced by geometrical parameters of weld profiles such as weld toe radius, reinforcement height, flank angle, weld throat thickness, and so on. During recent decades, systematical studies on the SCF of various types of joints, together with SCF parametric formulae were carried out by researchers. Iida and Uemura [10] summarized the SCF formulae widely used in Japan: Ushirokawa and Nakayama [11] proposed a comprehensive formula by the finite element method (FEM) to estimate the SCF of butt-welded joints, T-shape joints, and cruciform joints in tension and bending. The empirical formula by Nishida [12] was adopted by Ushirokawa and Nakayama [11] to consider the influence of the flank angle on SCF. This formula in [11] was then developed by Tsuji [13] for non-load carrying T-shape joints and cruciform joints based on the boundary element method (BEM).

Over the same period, Lawrence et al. [14] and Yung et al. [15] provided a general form to describe the SCF of welds for various types of joints with corresponding coefficients. Additionally, a parametric formula for T-shape joints in tension and bending was proposed by Monahan [16] and developed subsequently by Brennan et al. [17] and Hellier et al. [18] to cover the effect of the width of welded attachment. Recently, the formula was further modified by Kiyak et al. [19] to estimate the SCF for geometrically symmetric (Double-V) and asymmetric (Sing-V) butt-welded joints. Furthermore, the investigations were extended to post-welding (e.g., burr grinding) welds by researchers [20,21]. Some mathematical models were employed as well to predict the SCF, such as the artificial neural network [22], without an explicit formula.

Currently, the dimensions of the weld beads can be easily measured by modern measurement and modeling techniques, such as a 3D digital microscope. Thus, the SCF at the notches can be estimated by inserting dimensions into these formulae. This undoubtedly highlights the importance of the precision of these formulae.

Butt-welded joints, as we know, are among the most widespread joint types used in the industry. The SCF at the toe of butt weld can be estimated by the parametric formulae in [11,19,23]. It is worth pointing out that most of these formulae have ignored the influence of the weld width on SCF (e.g., [19,23]). However, in this paper, the SCF is found sensitive to the weld width in the case of the butt joints with narrow welds. The geometric model for the parametric study also needs to be considered carefully. For example, the trapezoid model is commonly employed to investigate the influences of parameters on SCF (e.g., [19]), but some configuration of the weld bead cannot be achieved by this kind of flat weld profile model, despite all the parameters being inside the respective ranges (see Section 2.1). The defects of the existing studies mentioned here will undoubtedly lead to inaccurate results for certain cases. Moreover, the parametric formulae are limited to specific application ranges, and some of them are found to be imprecise for the cases which are out of or even inside the application ranges [19]. Therefore, further investigations are encouraged to improve the precision and to extend the application ranges of the SCF formulae for butt-welded joints.

Furthermore, besides the influence of the weld profile discussed above, fabrication tolerances lead to stress magnification as well. For example, the angular distortion due to the thermal heat–input effect is common in butt-welded joints. It will induce additional secondary bending stress in welds when the joint is subjected to tensile loading. Several formulae are recommended to estimate the SCF caused by angular distortion in butt-welded joints, such as the formula by Berge and Myhre [24] and by the International Institute of Welding (IIW) [25]. It is worth noting that these formulae are only valid for the case that the joint with pre-existing distortion is loaded directly. However, in the course of a tensile fatigue test on butt-welded joint, a clamping procedure during assembly straightens the specimen first before cyclic loading. Consequently, the clamping stress is introduced in the specimen acting as additional mean stress for subsequent stress cycles.

Although the clamping procedure eliminates the overall distortion of the joint, the bending stress (*σ_b_*) caused by the tensile force still can be detected in the subsequent tension step due to the local residual distortion in the region around the welds, but it is much smaller than that of the case with direct tension procedure. Therefore, further investigations are encouraged on the distorted butt-welded joints under fatigue test conditions, so as to improve the transferability of fatigue specimen results to components. Only a few concerned studies can be found currently in the literature. For example, analytical and experimental research on the SCF of joints with angular distortion was carried out by Xing and Dong [26] and Ottersbock et al. [27] for cruciform joints and T-shape joints, respectively. To our best knowledge, there are no available wide-spread solutions for the SCF and clamping-induced stress of distorted butt-welded joints under fatigue test conditions.

This paper aims to propose parametric formulae to accurately estimate the SCF and clamping-induced stress at the weld toe of distorted butt-welded joints under fatigue test conditions. Owing to the extensive adaptability, a geometrical model with a spline curve is determined first for the parametric study. Subsequently, based on a large set of 2D finite element (FE) analyses, the existing SCF formulae for undistorted butt-welded joints are examined and discussed. The influence of the weld width on SCF, which is commonly neglected or misestimated by existing solutions, is investigated comprehensively. Consequently, a corresponding limitation on the application range of weld width is suggested. The SCF formula by Kiyak et al. [19] is then modified to achieve higher precision for undistorted butt-welded joints.

The complete fatigue test flow for distorted butt-welded joints containing clamping and loading procedures is simulated as well with FEM under various configurations of parameters. As a result, the parametric formulae for the SCF and clamping-induced stress at the weld toe of distorted butt joints under fatigue test conditions are proposed. Last, the formulae proposed by this paper are tested and proved to be valid and precise with a large number of FE analyses whose parameters are generated stochastically and independently in the corresponding application ranges. Such results allow an accurate evaluation of the SCF and clamping-induced stress for the distorted butt-welded joints under fatigue test conditions.

## 2. Finite Element Analysis

### 2.1. Geometric Model

**Figure 1:**
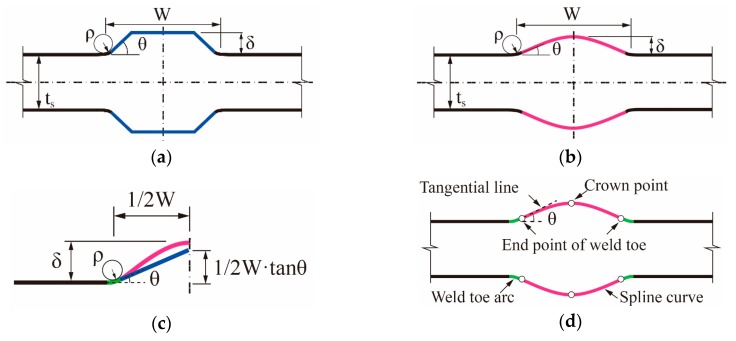
Geometric model of a butt-welded joint: (**a**) trapezoid model; (**b**) spline curve model; (**c**) implicit constraint of trapezoid model; and a (**d**) three-feature point spline curve model.

The trapezoid model (flat weld profile) and the spline curve model (rounded weld profile) are the two frequently-used geometric models for evaluating the SCF at the weld toes of butt-welded joints (see Figure 1a,b).

**Figure 2:**
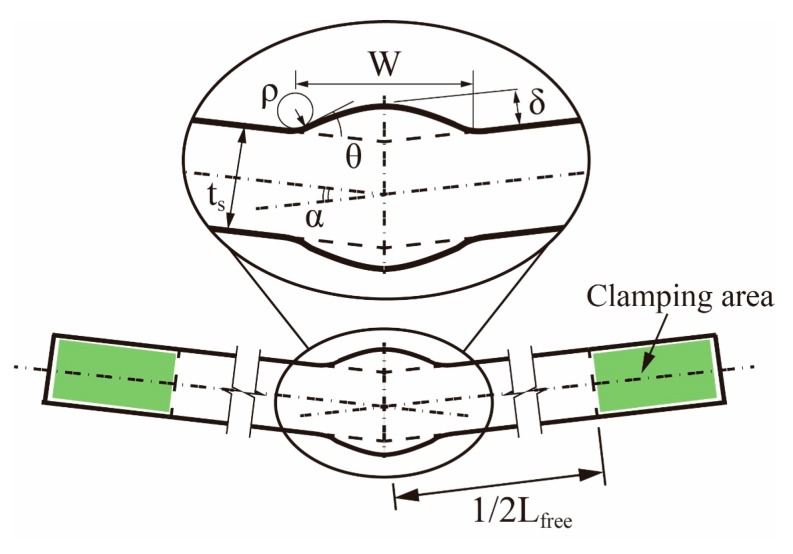
Parameter definition of distorted butt-welded joints under fatigue test conditions.

According to the comparison in [19], no significant difference in the first principal stress can be appreciated between the two options. However, Figure 1c shows the difference between the two models under the same flank angle (*θ*), which is defined as the intersection angle between the two tangential lines at the ends of the weld toe arc for the spline curve model. It can be seen that an implicit constraint on the application ranges of parameters is introduced by the trapezoid model: under a certain width of weld seam *W*, small flank angle *θ* with large reinforcement height (i.e., δ>1/2W⋅tanθ) is impossible to achieve. Contrarily, no such constraint exists in the spline curve model owing to its great adaptability. Therefore, the spline curve with three feature points is adopted in the present study to describe the profile of the weld bead (see Figure 1d). Idealized symmetric shape (Double-V) for the weld bead is assumed. The parameters, which affect the SCF at the weld toe of the joint, are illustrated in Figure 1b. The distortion angle (*α*) and the total length of the specimen (*L_free_*) between the hydraulic grips of a fatigue testing machine are also defined for distorted butt-welded joints (see Figure 2). The effective area of clamping is illustrated in Figure 2.

### 2.2. Finite Element Model

#### 2.2.1. Finite Element Model for Undistorted Butt-Welded Joints

**Figure 3:**
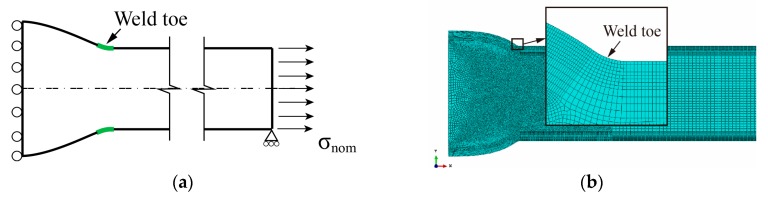
Finite element (FE) model for an undistorted butt-welded joint: (**a**) sketch of one-half model; (**b**) typical global and local meshes.

A one-half model with a symmetrical boundary condition is employed to calculate the SCF at the weld toe of butt joints (see Figure 3a). Uniform tensile stress is applied to the extremity of the plate acting as the nominal (membrane) stress (*σ_nom_*). The first principal stress at the weld toe surface (nodal result) is taken for the notch stress (*σ_notch_*). Consequently, the elastic notch SCF *K_t_* at the weld toe can be defined as shown in Equation (1):

(1) $$K_{t}=\sigma_{notch}/\sigma_{nom}.$$

**Figure 4:**
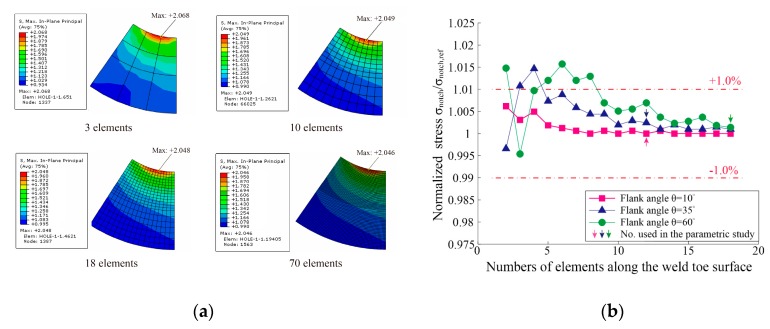
Convergence analysis of the mesh: (**a**) the first principal stresses under different numbers of elements: flank angle $\theta =35^{\circ }$; (**b**) normalized stresses under different numbers of elements.

Isotropic linear elastic material with elastic modulus $E_{s}=210$ GPa and Poisson’s ratio n=0.3 is assumed. All finite element models have been built up with 2D-solid plane strain elements in Abaqus CAE version 6.13-1 [28]. Eight-node biquadratic fully integrated quadrilateral elements with nine integration points are chosen (ABAQUS element name: CPE8). Very fine meshes are employed to ensure the computational accuracy of various configurations: more than 12 elements are assigned along the weld toe surface, the aspect ratios for the edges of the elements are about 1.0 which is slightly fluctuant due to the rounding of the number of elements in the radial direction. The typical global and local meshes are shown in Figure 3b. According to the convergence analysis of mesh by Baumgartner and Bruder [29], the notch stresses of butt welds in terms of the first principal stress converge quickly towards the final value. Only 20 elements over 360 degrees (i.e., four elements over 60 degrees) with a 1.0 aspect ratio results in an error less than 2.5%. Nevertheless, the convergence of the mesh is tested in this paper before the parametric study. The first principal stresses at the notch surface *σ_notch_* are calculated under distinct element numbers. Figure 4a shows the typical results of the case with flank angle $\theta =35^{\circ }$. All calculated notch stresses are normalized by reference stresses *σ_notch,ref_* which are calculated with FE models having a very fine mesh (two elements per degree). The determined normalized stresses *σ_notch_/σ_notch,ref_* are plotted in Figure 4b, the saw tooth characteristic of the results comes from differing locations of the maximum stress [29]. It can be seen that, for using CPE8 elements to calculate the first principal stress at the notch of a butt joint, the error is less than 2% even with only two elements over 60 degrees (the maximum flank angle considered in the current study). Hence, the mesh in this paper is fine enough to provide accurate results for the parametric study.

#### 2.2.2. Finite Element Model for Distorted Butt-Welded Joints

**Figure 5:**
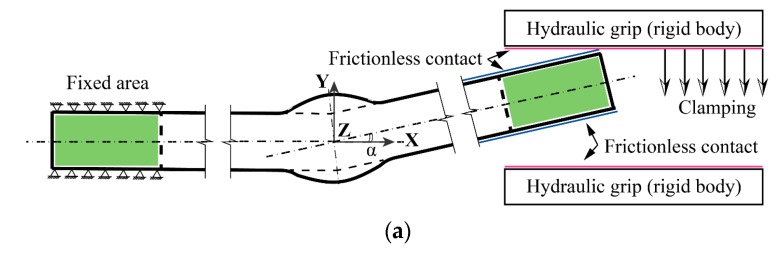

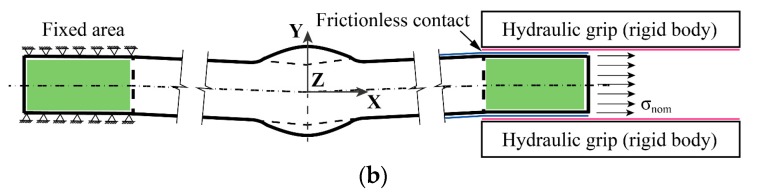
Two-step FE analysis for a distorted butt joint: (**a**) clamping; (**b**) tension.

When it comes to distorted butt-welded joints, the simulation should be consistent with the course of the fatigue test as much as possible to catch the SCF and clamping-induced stress at the weld toe of the joint. Hence, a two-step FE analysis with the same material and mesh parameters as undistorted butt-welded joints (see Section 2.2.1) is carried out for distorted joints under fatigue test conditions, as illustrated in Figure 5. In detail, the left clamping area is fully fixed corresponding to a specimen gripped on one side [27]. In the first step, hydraulic grips, which are modelled with rigid bodies, straighten the distorted joints on the other side. Subsequently, uniform tensile stress (*σ_nom_*) is applied to the extremity of the plate after clamping. Frictionless contact between the grip and plate is assumed.

### 2.3. Application Ranges of Parameters

As discussed earlier, the precision of the SCF formulae should be evaluated with corresponding application ranges. The SCF at the weld toe does not depend on the absolute values of parameters but the relative ratios. Hence, wide normalized parameter ranges were determined for the subsequent parametric formulae as shown below:

- Weld toe radius *ρ/t_s_*: 0.01–0.40;
- Weld reinforcement height *δ/t_s_*: 0.05–0.40;
- Width of weld seam *W/t_s_*: 1.0–2.0;
- Flank angle *θ*: 10°–60°;
- Distortion angle *α*: 0–3°; and
- Total length of specimen *L_free_/t_s_*: 10.0–40.0.

The same parameter ranges as Kiyak et al. [19] for the weld profile (i.e., *ρ/t_s_*, *δ/t_s_*, and *θ*) are considered here. An additional limitation on the range of weld width (*W*) is supplemented based on numerous FE results (discussed in Section 3.2) to ensure the precision of the SCF parametric formula. Reasonable ranges for the distortion angle (*α*) and the total length of specimen (*L_free_*) are determined as well.

## 3. Elastic SCF Formulae for Undistorted Butt-Welded Joints

### 3.1. Overview on Existing K_t_ Formulae

As referred to in [19], many of the existing parametric formulae have not been developed especially for the butt-welded joints, but, for other types, such as T-shape joints and cruciform joints. Still, several studies that focus on butt joints can be found in the literature as displayed in Table 1. The corresponding formulae are summarized in Appendix A.

**Figure 6:**
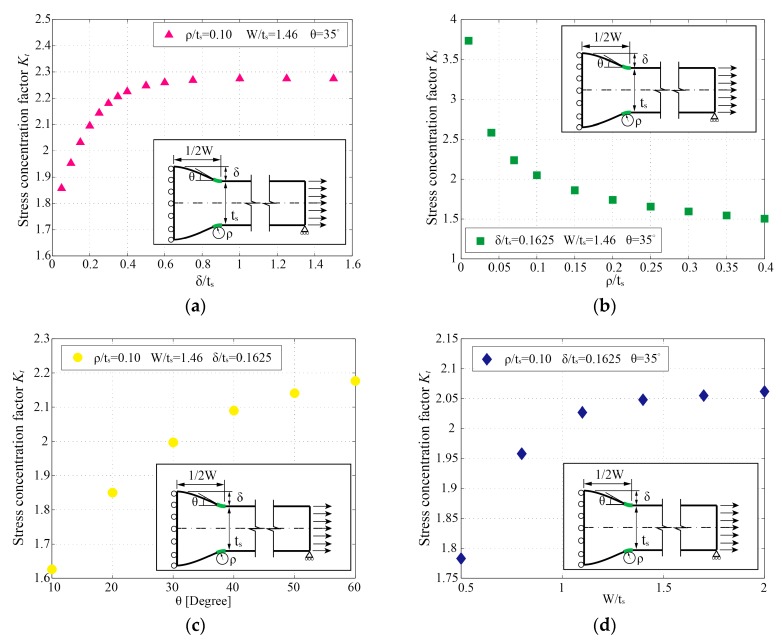
The influences of parameters on the stress concentration factor (SCF) (*K_t_*): (**a**) reinforcement height; (**b**) weld toe radius; (**c**) flank angle; (**d**) weld width.

It is worth noting that, according to the referenced conclusion in [19] that originated from two German masters theses (refer to [19] p. 1248 for more information), the SCF formulae for T-shape joints and cruciform joints can be applied to estimate the SCFs of geometrically symmetric (Double-V) and asymmetric (Single-V) butt-welded joints, respectively. This conclusion is, to some extent, applicable for basic estimation. Figure 6a displays the influence of the reinforcement height (*δ*) on the SCF (*K_t_*) in a Double-V butt-welded joint. Essentially, with the increase of *δ*, the growth rate of *K_t_* decreases gradually to zero. The geometric profile of cruciform joints can be simply regarded as butt joints with large *δ*. Hence, the *K_t_* of butt joints is likely to be overestimated by the formulae for T-joints or cruciform joints if no correction is further considered. Nevertheless, the SCF formulae for T-joints in [13,16,17,18] are still summarized and compared by Kiyak et al. [19]. The results indicate that the extended *K_t_* formula proposed by Kiyak et al. [19] (see Equation (A2)) for butt-welded joints gains the highest precision. Additionally, the influences of the weld toe radius (*ρ*), flank angle (*θ*), and weld width (*W*) are plotted in Figure 6b–d, respectively.

**Figure 7:**
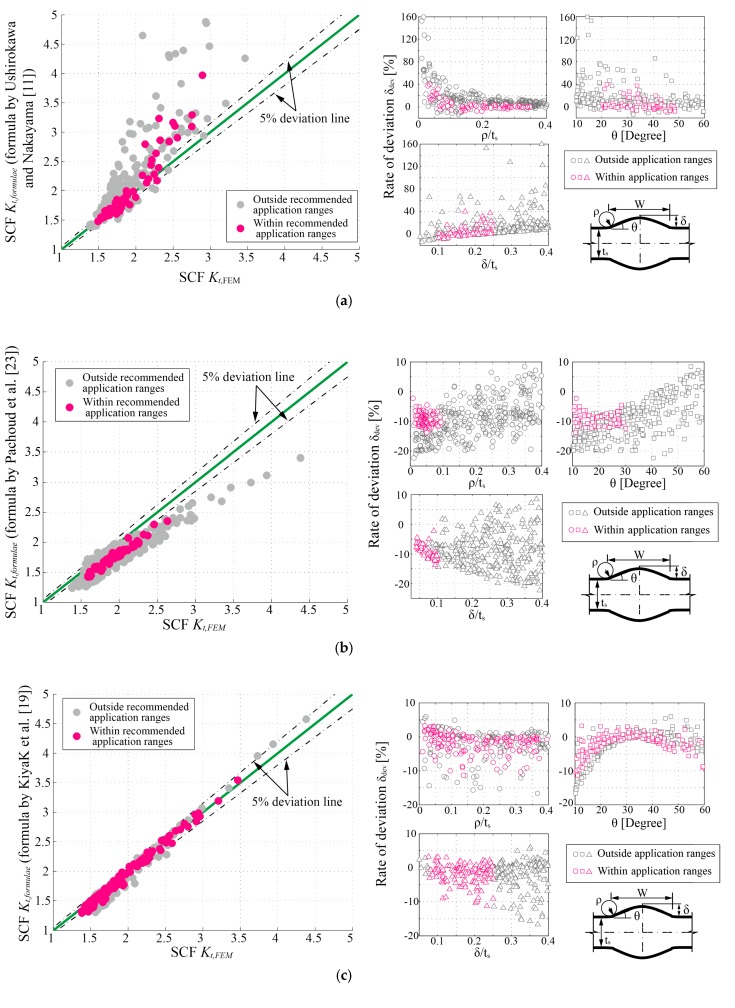
Comparisons of the SCF (*K_t_*) determined by parametric formulae and the finite element method (FEM): (**a**) Ushirokawa and Nakayama [11]; (**b**) Pachoud et al. [23]; (**c**) Kiyak et al. [19].

To examine the validity of previous formulae listed in Table 1, 305 FE models were made and calculated according to the approach mentioned in Section 2.2.1. For these models, each parameter was stochastically and independently selected within its own application range (defined in Section 2.3). As a result, the comparisons between the *K_t_* determined by FEM and previous parametric formulae are plotted in Figure 7a–c. The results are divided into two groups based on whether they are within the respective recommended application ranges. The rate of deviation *δ_dev_*, which is defined in Equation (2), is also plotted in Figure 7a–c to clarify the source of the errors:

(2) $$\delta_{\mathrm{dev}}=\left(K_{t,formula}-K_{t,FEM}\right)/K_{t,FEM},$$

where *K_t,formulae_* and *K_t,FEM_* represent the SCF determined by parametric formulae and FE analysis, respectively.

Overall, the formula by Ushirokawa and Nakayama [11] provides conservative solutions. It performs well in the low *K_t_* area (i.e., $K_{t}=1.5\sim 2.0$), while the deviations increase with the rising of *K_t_*. According to the results of the deviation analysis in Figure 7a, the rates of deviations increase sharply with the decreasing of the weld toe radius (*ρ/t_s_*). Moreover, as the increasing of reinforcement height (*δ/t_s_*), the deviations show an upward trend. Conversely, the formula proposed by Pachoud et al. [23] gives overall nonconservative results but higher precision than those in [11]. For some reason, it always seems to be about 10% smaller than the results calculated by this paper. The formula by Kiyak et al. [19] still has the best performance and the widest application ranges among the three. However, the deviations show a strong correlation with the flank angle (*θ*); this phenomenon is most likely due to the implicit constraint between the flank angle (*θ*) and reinforcement height (*δ*) of the trapezoid model. The available range of flank angle (*θ*) may be constrained by the reinforcement height (*δ*) under a certain weld width (*W*) (see Section 2.1) during their parametric study.

### 3.2. The Influence of Weld Width

To reduce the calculation cost, some parameters are usually kept constant by previous research during parametric studies. For example, Tsuji [13] fixed the flank angle (*θ*) of T-shape joints to 45°; only two values of reinforcement height (*δ*) are considered by Kiyak et al. [19]. In these cases, the applicability of the formulae to these parameters should be specially examined. Kiyak et al. [19] proved that their formula can be used within the range *δ/t_s_*: 0.075–0.25. However, when it comes to the width of the weld seam (*W*), which is fixed to $W/t_{s}=1.46$ in [19], has not been considered in their formula, nor discussed. As a consequence, imprecise solutions can be found under distinct *W/t_s_*. Differently, the influence of *W* on *K_t_* has been studied by Pachoud et al. [23] within the range *W/t_s_*: 0.58–1.19 and been considered to be inconspicuous. Thus, the parameter *W* is neglected by the formula in [23] as well. However, only one set of parameter combinations was calculated by [23]; this may lead to a one-sided conclusion. In addition, the effect of *W* has been considered in the formulae by Ushirokawa and Nakayama [11], but the predicted *K_t_* results under distinct *W*, as well as the variation tendencies of *K_t_* with *W* being inconsistent with the FE results by this paper.

**Figure 8:**
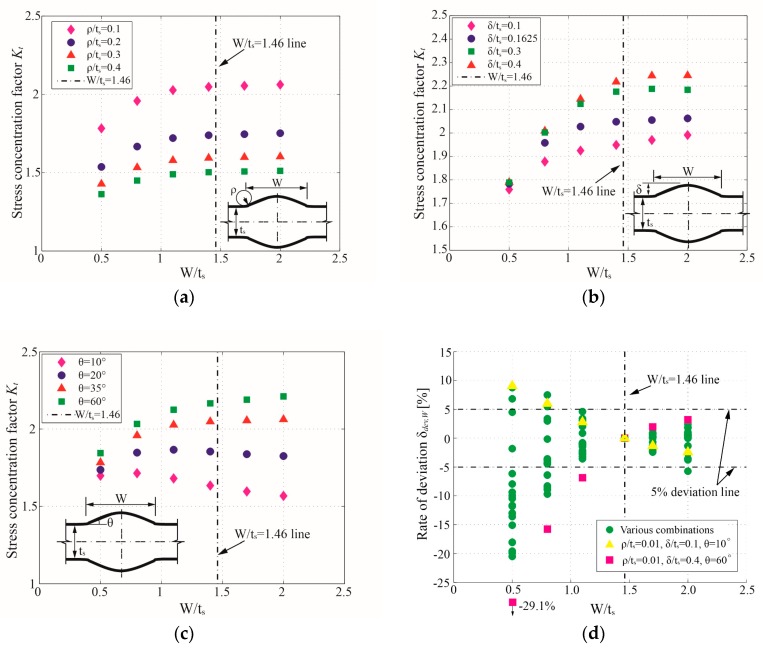
The influence of the weld width (*W*) on SCF (*K_t_*): (**a**) FE results under various *ρ/t_s_*, constant parameters: $\delta /t_{s}=0.1625$ and $\theta =35^{\circ }$; (**b**) FE results under various *δ/t_s_*, constant parameters: $\rho /t_{s}=0.1$ and $\theta =35^{\circ }$; (**c**) FE results under various *θ*, constant parameters: $\rho /t_{s}=0.1$ and $\delta /t_{s}=0.1625$; (**d**) deviation rates of *K_t_* under various *W/t_s_* compared to $K_{t,W/t_{s}=1.46}$.

To comprehensively evaluate the effect of *W* on *K_t_*, a series of FE analyses were executed by this paper under the combinations of *W/t_s_* and *ρ/t_s_*, *δ/t_s_*, and *θ* (see Figure 8a–c). The SCF under $W/t_{s}=1.46$ ($K_{t,W/ts=1.46}$), which is assumed in [19], is marked in each figure. We noticed that, except for some cases with a small flank angle (e.g., $\theta =10^{\circ }$), the *K_t_* of butt joints decrease with the decrease of *W*. The deviation rates of *K_t_* (*δ_dev,W_*) caused by fixing *W/t_s_* to 1.46 are calculated with Equation (3) and plotted in Figure 8d:

(3) $$\delta_{dev,W}=\left(K_{t}-K_{t,W/t_{s}=1.46}\right)/K_{t,W/t_{s}=1.46}.$$

From Figure 8d, some unsatisfactory deviation rates ($\delta_{dev,W}\geq 5\%$) are detected when $W/t_{s}\leq 1.0$. More specifically, *K_t_* is visibly overestimated by $K_{t,W/t_{s}=1.46}$ in most cases when $W/t_{s}\leq 1.0$, except for some cases with small *θ* (e.g., $\theta =10^{\circ }$), which has an opposite conclusion. Therefore, in order to ensure the precision of the *K_t_* formula, a supplementary limitation on *W* is proposed as *W/t_s_*: 1.0–2.0 in the present study. 

### 3.3. K_t_ Parametric Formula

**Figure 9:**
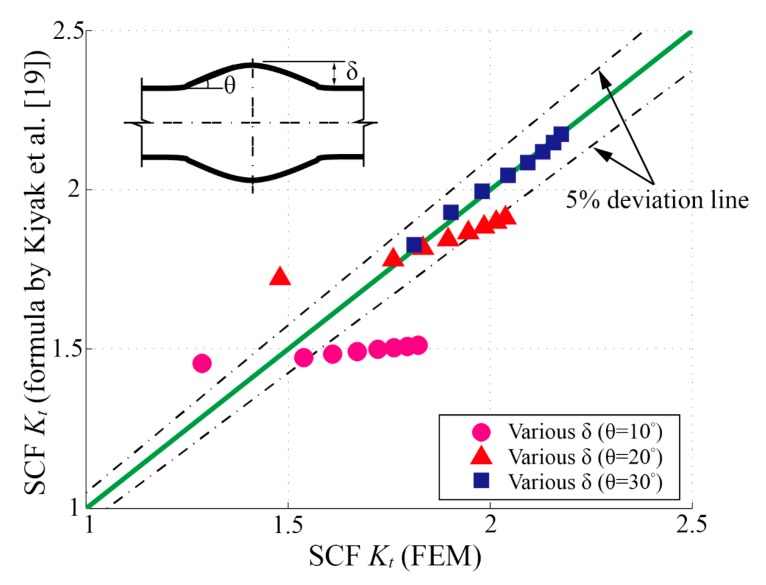
Comparison of the stress concentration factor (SCF) (*K_t_*) determined by finite element method (FEM) and the parametric formula in [19] under the small *θ* cases, reinforcement height: $\delta /t_{s}=0.05\sim 0.4$.

As shown in Figure 7c, the formula by Kiyak et al. [19] performs well on *K_t_* evaluation within a wide application range. In addition to supplying the limitation on the application range of *W* as discussed above, further modification on this formula should be implemented as well to fix the defect originated from the trapezoid geometric model as discussed earlier in Section 2.1. Although the formula by Kiyak et al. [19] extends the application range to an angle as small as 10°, some profiles of weld beads, which cannot be achieved by the trapezoid model (i.e., tanθ<2δ/W) but do exist in reality, are not covered by their formula. A simple check with the combinations of *θ* and *δ/t_s_* is carried out to clarify this problem; see Figure 9. Due to the implicit constraint introduced by the trapezoid model (see Figure 1c), the application range of flank angle *θ* is confined by the reinforcement height *δ*. Hence, the formula by Kiyak et al. [19] performs unsatisfactorily in its application ranges when *θ* is relatively small (e.g., $\theta =10^{\circ },20^{\circ }$).

Therefore, 280 sets of FE analyses, which contain a mass of cases with small *θ*, were implemented by the present paper with various combinations of parameters. The *K_t_* results determined by FEM were adopted as training data to further fitting the formula by Kiyak et al. [19]. Consequently, a new *K_t_* parametric formula for geometrically symmetric (Double-V) butt-welded joints loaded in tension is carried out based on the form by Kiyak et al. [19] (see Equation (4)):

(4) $$K_{t}=1+p_{1}{\left(\frac{\delta }{t_{s}}\right)}^{p_{2}\cdot \theta }\cdot \theta^{p_{3}}\cdot e^{-p_{4}\cdot \theta }\cdot {\left(\frac{\rho }{t_{s}}\right)}^{-0.288\cdot \theta }\cdot {\left(0.014+\frac{\rho }{t_{s}}\right)}^{-p_{5}}\cdot \left(p_{6}\cdot {\left(\frac{\delta }{t_{s}}\right)}^{2}+p_{7}\cdot \left(\frac{\delta }{t_{s}}\right)+p_{8}\right),$$

with the coefficients given in Table 2 (*e* represents Euler’s number). The flank angle *θ* and distortion angle *α* in the formulae of this paper are both in the unit of radians.

**Figure 10:**
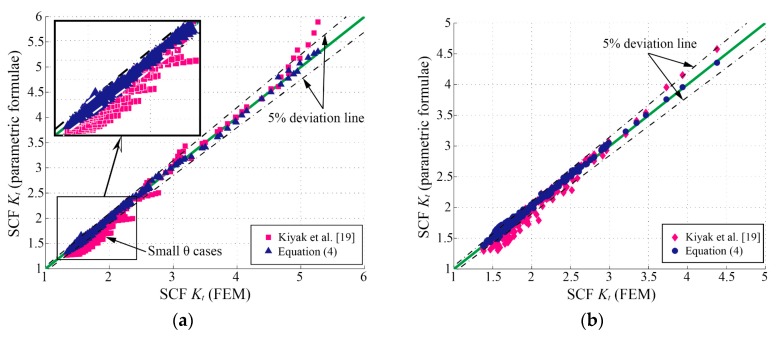
Comparisons of the SCF (*K_t_*) determined by FEM and parametric formulae: (**a**) training data system; (**b**) test data system.

Figure 10a shows the comparisons between the *K_t_* determined by FEM and parametric formulae in the training data system. Equation (4) provides more accurate results than the formula by Kiyak et al. [19] as compared to FEM results.

As mentioned in Section 3.1, the 305 sets of *K_t_* results obtained from the FE analyses, whose parameters are selected stochastically and independently in the application ranges, were employed as test data to examine the validity of Equation (4) (see Figure 10b). As expected, Equation (4) performs better than the formula by Kiyak et al. [19] and is proved to be valid and accurate in the wide application range.

## 4. Elastic SCF and Clamping Stress Formulae for Distorted Butt-Welded Joints

### 4.1. Definition of K_m,test_ and K_act_

**Figure 11:**
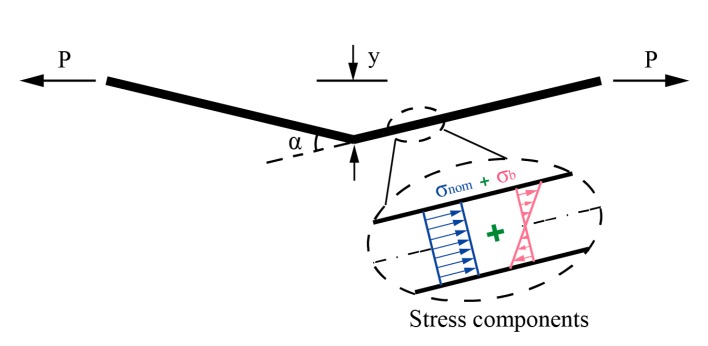
Stress components of the plates with angular misalignment under the axial tension.

Section 3 has addressed the SCF for undistorted butt-welded joints, and the influence of the angular distortion on the SCF will be discussed in this section. As mentioned earlier, the fatigue performance of welded joints can be significantly influenced by the angular distortion which introduces secondary bending at the presence of axial tension on the joints. Figure 11 displays the angular misalignment between two flat plates. Under the axial tension, an additional bending moment is introduced in the plate. The bending moment acting at the intersection can be obtained by multiplying the tensile force *P* and the distance *y* (see Figure 11). Consequently, besides the nominal stress *σ_nom_* (membrane stress), the additional bending stress *σ_b_* also can be detected in the plate as shown in Figure 11. 

Hence, the local stress at the weld toe of distorted butt joints consists of the nominal stress *σ_nom_* and bending stress *σ_b_*. The SCF contributed by angular distortion (*K_m_*) is defined as shown in Equation (5):

(5) $$K_{m}=\frac{\sigma_{nom}+\sigma_{b}}{\sigma_{nom}}=1+\frac{\sigma_{b}}{\sigma_{nom}}.$$

Both *σ_nom_* and *σ_b_* are magnified by *K_t_* (the SCF caused by weld profile, see Equation (4)). Therefore, the total stress *σ_notch_* at the weld toe of distorted joints can be expressed as shown in Equation (6):

(6) $$\sigma_{notch}=K_{t}\cdot \left(\sigma_{nom}+\sigma_{b}\right).$$

Thus, the total SCF (*K_t,m_*) for distorted butt joints can be derived as shown in Equation (7):

(7) $$K_{t,m}=\frac{\sigma_{notch}}{\sigma_{nom}}=\frac{K_{t}\cdot \left(\sigma_{nom}+\sigma_{b}\right)}{\sigma_{nom}}=K_{t}\cdot K_{m}.$$

**Figure 12:**
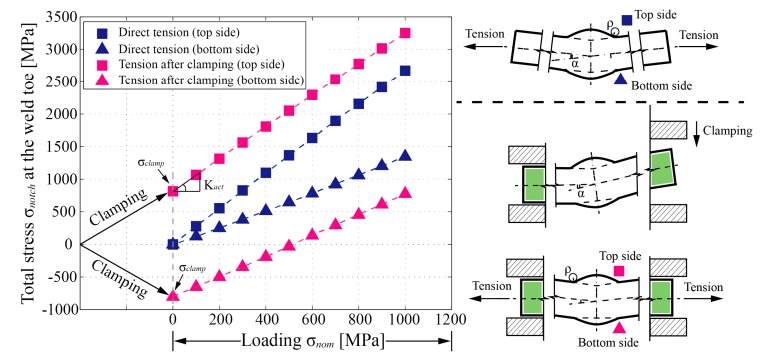
Total stress (the third principal stress for negative values, the first principal stress for positive values) at the weld toe of distorted butt-welded joints under two different loading processes, parameters: $\rho /t_{s}=0.1$, $\delta /t_{s}=0.1625$, $W/t_{s}=1.0$, $\theta =35^{\circ }$, $\alpha =3^{\circ }$, $L_{free}/t_{s}=12.7$.

Several formulae can be employed to quantitatively estimate the SCF caused by angular distortion (Km), such as the formulae provided by Berge and Myhre [24] and IIW [25]. These formulae are built for the case that the joints with pre-existing distortion are loaded in tension directly. They cannot be applied to the distorted specimens under fatigue test conditions. In the case of fatigue tests, the distorted butt joint must be mounted to a fatigue testing machine. The joint will be straightened first by the hydraulic gripping system (see Figure 5a). As a result, initial stress fields are introduced in the joint. The stress at the weld toe, which is introduced by this clamping procedure, is defined as clamping stress *σ_clamp_*. Subsequently, a tensile loading is applied to the joint after clamping (see Figure 5b). Although the clamping procedure in the previous step has eliminated the overall distortion, the bending stress *σ_b_* generated by the tensile force (rather than by the clamping procedure) still can be detected in this tension step due to the local residual distortion in the region around the welds, but it is much smaller than that of the case with direct tension. Figure 12 quantitatively illustrates the difference between the two loading processes mentioned here: direct tension and tension after clamping.

Essentially, the growth rate of the total notch stress *σ_notch_* with loading stress *σ_nom_* in Figure 12 is the total SCF (*K_t,m_*) of the butt joints that has been defined in Equation (7). The influence of the clamping stress *σ_clamp_* should be excluded when calculating the total SCF for the case with tension after clamping (i.e., fatigue test condition). To distinguish between the two different loading processes in Figure 12, the actual total SCF for the case with tension after clamping is represented by the symbol *K_act_*. As compared to *K_t,m_* for the direct tension case, *K_act_* can be expressed as shown in Equation (8):

(8) $$K_{act}=K_{t}\cdot K_{m,test}=\frac{\sigma_{notch}-\sigma_{clamp}}{\sigma_{nom}},$$

where *K_m,test_*, as compared to *K_m_* for the direct tension case, represents the SCF contributed by residual angular distortion after clamping. It also can be expressed by Equation (5), but here the bending stress *σ_b_* is only originated from the residual angular distortion.

It is worth noting that the relationship between the total notch stress *σ_notch_* and loading stress *σ_nom_* is supposed to be nonlinear. However, after carefully checking, the same result as [26] is concluded that an approximately linear relationship can be considered for the cases with tension after clamping (i.e., fatigue test condition). Therefore, in the present paper, the total notch stress *σ_notch_* at the weld toe under 500 MPa tensile stress is employed for *σ_notch_* when calculating the actual SCF (*K_act_*) by Equation (8).

Only a few studies have considered the SCF (*K_act_*) under fatigue test conditions. Ottersbock et al. [27] recently proposed a formula to describe the functional relationship between the actual SCF *K_act_* of distorted T-shape joints and distortion angle *α*, tensile loading *σ_nom_*. The formula mainly contributes to the hot-spot stress approach and the effective notch stress approach, both of which use the simplified profile of weld bead (e.g., the effective notch stress approach assumes 1.0 mm toe radius with 45° flank angle for the welds of T-shape joints). Therefore, the variety of weld profile parameters (e.g., *ρ*, *θ*, and *W*) and loading conditions (e.g., *L_free_*) are not considered in the formula. Xing and Dong [26] presented a series of formulae to estimate the SCF in a cruciform joint containing either axial or angular misalignment based on a general analytical method. The study by Xing and Dong [26] is also extended to fatigue test conditions. The two steps in the fatigue test including clamping and tension are analyzed respectively. As a result, the formula to estimate the *K_m,test_* of distorted cruciform joints is provided as shown in Equation (A4).

To the best of our knowledge, there are no solutions available in the literature to estimate the clamping stress *σ_clamp_* at the weld toe of butt joints. Although Ottersbock et al. [27] have proposed a set of formulae to evaluate the *σ_clamp_* of distorted T-shape joints in terms of nominal stress, hot-spot stress and notch stress, these formulae mainly contribute to clarify the linear relationship between *σ_clamp_* and *α*. Only distortion angle *α* was considered as the independent variable in the formulae. To cover all the influences of parameters (i.e., *ρ/t_s_*, *δ/t_s_*, *W/t_s_*, *θ*, *α*, and *L_free_*), a comprehensive *σ_clamp_* parametric formula is encouraged.

### 4.2. K_m,test_, K_act_, and σ_clamp_ Parametric Formulae

To obtain the parametric formulae that can be used to evaluate the SCF under fatigue test conditions (i.e., tension after clamping), a series of two-step FE analyses (discussed in Section 2.2.2) were implemented under the combinations of distortion angle *α* and the total length of specimen *L_free_* in respective application ranges (defined in Section 2.3). Accordingly, a series of *K_m,test_* were calculated by Equation (5) with the bending stress *σ_b_* (contributed by the residual angular distortion after clamping procedure) extracted from FEM results. These results were employed as training data to fit the parametric formula for *K_m,test_*. As a result, the empirical *K_m,test_* formula was carried out as shown in Equation (9):

(9) $$K_{m,test}=1+A_{1}\cdot \alpha \cdot \left(\ln\left(\frac{L_{free}}{2\cdot t_{s}}\right)+A_{2}\right),$$

with the coefficients given in Table 3. It is worth noting that both *K_m,test_* and *K_act_* derived by this paper are positive at the weld toe on the top side as indicated in Figure 12.

**Figure 13:**
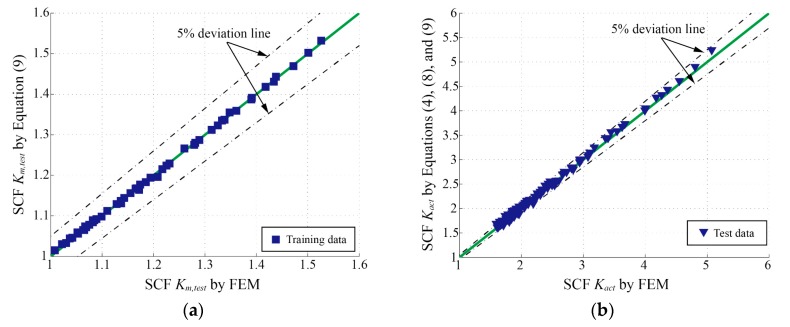
Comparison of the SCF determined by FEM and parametric formulae: (**a**) comparison of *K_m,test_* in training data system; (**b**) comparison of *K_act_* in test data system.

Figure 13a shows the comparisons between the *K_m,test_* determined by FEM and Equation (9) in training data system. As expected, Equation (9) performs well on predicting the *K_m,test_*.

Following this, *K_act_* parametric formula can be composed of Equations (4), (8) and (9). In order to examine the validity of the *K_act_* formula proposed by this paper, 130 FE models were made and calculated according to the approach mentioned in Section 2.2.2. Similarly, for these models, each parameter was stochastically and independently selected within its own application range (defined in Section 2.3) including the parameters of weld bead profile (i.e., *ρ/t_s_,* *δ/t_s_, W/t_s_,* and *θ*) and angular distortion (i.e., *α*, and *L_free_*). The corresponding *K_act_* derived from Equation (8) with the stresses *σ_notch_* and *σ_clamp_* extracted from FEM results were used as test data. Figure 13b shows the comparison between the *K_act_* determined by FEM and parametric formulae proposed by this paper in test data system. As expected, the *K_act_* parametric formula, which is composed of Equations (4), (8) and (9) by this paper, can precisely evaluate the total SCF for the distorted joint under fatigue test conditions in a wide range of application.

Undoubtedly, the clamping stress *σ_clamp_* at the weld toe has a functional relationship with the weld toe SCF *K_act_*. Therefore, a series of clamping stresses *σ_clamp_*, which result from the two-step FE analyses mentioned above, are also employed as training and test data for fitting the *σ_clamp_* parametric formula. Consequently, the empirical *σ_clamp_* formula is carried out as shown in Equation (10):

(10) $$\sigma_{clamp}(MPa)=K_{act}\cdot \left(B_{1}\cdot \alpha^{B_{2}}\cdot {\left(\frac{L_{free}}{2\cdot t_{s}}\right)}^{B_{3}\cdot \alpha +B_{4}}\right),$$

with the coefficients given in Table 4. The *K_act_* in Equation (10) can be calculated by Equations (4), (8), and (9). The obtained *σ_clamp_* here is the first principal stress at the weld toe on the top side as indicated in Figure 12.

**Figure 14:**
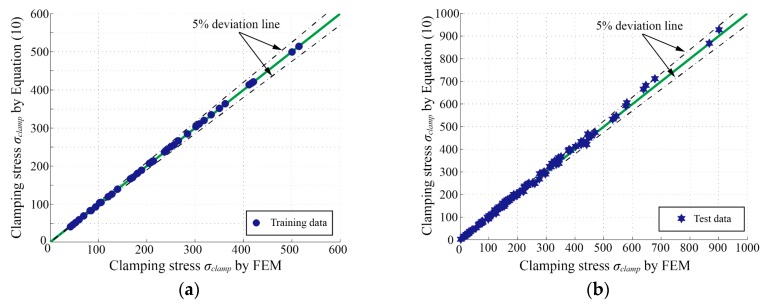
Comparisons of the clamping stresses (*σ_clamp_*) determined by FEM and Equation (10): (**a**) training data system; (**b**) test data system.

Figure 14a,b show the comparisons between the *σ_clamp_* determined by FEM and Equation (10) in the training data system and test data system, respectively. As expected again, the precision of Equation (10) is satisfactory enough.

## 5. Conclusions

In this paper, parametric studies on the elastic SCF at the weld toe of undistorted and distorted butt-welded joints under tensile fatigue test conditions were executed based on a large set of 2D FE analyses. A wide application range of parameters were considered as follows:

- Weld toe radius *ρ/t_s_*: 0.01–0.40;
- Weld reinforcement height *δ/t_s_*: 0.05–0.40;
- Width of weld seam *W/t_s_*: 1.0–2.0;
- Flank angle *θ*: 10°–60°;
- Distortion angle *α*: 0–3°; and
- Total length of specimen *L_free_/t_s_*: 10.0–40.0.

*K_t_* and *K_m,test_* formulae were proposed to evaluate the SCF caused by weld bead geometry and residual angular distortion (after the clamping procedure), respectively. Consequently, the formula of total SCF *K_act_* under fatigue test conditions was obtained by multiplying *K_t_* and *K_m,test_*. The *σ_clamp_* formula, which can be used to estimate the clamping-induced stress at the weld toe of distorted butt joints in fatigue tests, was proposed as well. All of these parametric formulae were determined and examined with numerous training data and test data, respectively. Relevant existing solutions in the literature were reviewed and compared, and the results of the comparisons indicate that the proposed formulae in this paper provide the best estimates. These formulae can be used for fatigue life estimation based on local stress-life approaches, for calculating the actual effective stress ratio at the weld toe of distorted butt-welded joints under the cyclic loading of fatigue test.

## Figures and Tables

**Table 1:** Elastic stress concentration factor (SCF) (*K_t_*) parametric formulae for butt-welded joints loaded in tension.

Approach	Parametric Formula	Application Range
Ushirokawa and Nakayama [11]	Equation (A1)	*ρ/t_s_*: 0.025–0.35 *δ/t_s_*: 0.1–0.25 *W/t_s_*: 0.5 *θ*: 20°–50°
Kiyak et al. [19]	Equation (A2)	*ρ/t_s_*: 0.01–0.4 *δ/t_s_*: 0.075; 0.25 ^1^ *W/t_s_*: 1.46 *θ*: 10°–60°
Pachoud et al. [23]	Equation (A3)	*t_s_*: 20; 35; 50 mm *ρ*: 0.4–1.9 mm *δ/t_s_*: 0.04–0.1 *W/t_s_*: 0.58–1.19 ^2^ *θ*: 5°–30°

^1^ Only two values of reinforcement height *δ/t_s_* have been considered in [19], but the formula is checked and recommended to use within the range *δ/t_s_*: 0.075–0.25 (refer to [19] for more information), ^2^ derived from the range of edge preparation angle in [23].

**Table 2:** Coefficients in Equation (4).

**Coefficient**	$p_{1}$	$p_{2}$	$p_{3}$	$p_{4}$	$p_{5}$	$p_{6}$	$p_{7}$	$p_{8}$
**Value**	1.398	−0.144	0.715	1.650	0.322	−2.233	2.319	0.526

**Table 3:** Coefficients in Equation (9).

**Coefficient**	$A_{1}$	$A_{2}$
**Value**	5.582	−1.200

**Table 4:** Coefficients in Equation (10).

**Coefficient**	$B_{1}$	$B_{2}$	$B_{3}$	$B_{4}$
**Value**	56,476.872	0.992	−2.208	−1.080

## Appendix A

Ushirokawa and Nakayama [11], the SCF formula for butt-welded joints loaded in tension:

(A1) $$\begin{array}{c} K_{t}=1+\frac{1-\exp\left(-0.90\cdot \theta \cdot \sqrt{\frac{P}{2\delta }}\right)}{1-\exp\left(-0.90\cdot \left(\frac{\pi }{2}\right)\cdot \sqrt{\frac{P}{2\delta }}\right)}\cdot 2{\left(\frac{\delta }{\rho }\cdot \frac{1}{2.8\left(\frac{P}{t_{s}}\right)-2}\right)}^{0.65},\\ P=t_{s}+2\delta +0.6W. \end{array}$$

Kiyak et al. [19], the SCF formula for butt-welded joints loaded in tension (Double-V):

(A2) $$K_{t}=1+1.9220{\left(\frac{\delta }{t_{s}}\right)}^{0.3224\cdot \theta }\cdot \theta^{1.1257}\cdot e^{-1.5481\cdot \theta }\cdot {\left(\frac{\rho }{t_{s}}\right)}^{-0.295\cdot \theta }\cdot {\left(0.021+\frac{\rho }{t_{s}}\right)}^{-0.4002}.$$

Pachoud et al. [23], the SCF formula for butt-welded joints loaded in tension:

(A3) $$K_{t}=1+0.81{\left(\frac{\delta }{t_{s}}\right)}^{0.11}{\left(\frac{\rho }{t_{s}}\right)}^{-0.40}\tan{\left(\frac{\theta }{2}\right)}^{0.59}.$$

Xing and Dong [26], the *K_m,test_* formula for distorted cruciform joints under fatigue test conditions:

(A4) $$K_{m,test}=1+\alpha \cdot \left(\frac{4}{5}\cdot \frac{L_{free}^{4}-9L_{free}^{3}L_{c}+39L_{free}^{2}L_{c}^{2}-60L_{c}^{3}L_{free}+30L_{c}^{4}}{L_{free}^{3}\cdot t_{s}}\right),$$

where *L_c_* is the length from the hydraulic grip to the position of critical location, and it can be determined by Equation (A5):

(A5) $$L_{c}=\frac{1}{2}\left(L_{free}-W\right),$$

and the *W* here represents the width of the attachment in a cruciform joint.
